# ESBL- and Carbapenemase-Producing *Escherichia coli* and *Klebsiella pneumoniae* among Bivalves from Portuguese Shellfish Production Areas

**DOI:** 10.3390/microorganisms11020415

**Published:** 2023-02-07

**Authors:** Samanta Freire, Teresa Grilo, Bruna Rodrigues, Rui Oliveira, Carla Esteves, António Marques, Laurent Poirel, Marta Aires-de-Sousa

**Affiliations:** 1 Laboratory of Molecular Biology, Portuguese Red Cross, 1600-680 Lisboa, Portugal; 2 Instituto Português do Mar e Atmosfera, 1495-165 Lisboa, Portugal; 3 Medical and Molecular Microbiology Unit, Faculty of Science and Medicine, University of Fribourg, 1700 Fribourg, Switzerland; 4 INSERM European Unit (IAME, France), University of Fribourg, 1700 Fribourg, Switzerland; 5 Swiss National Reference Center for Emerging Antibiotic Resistance (NARA), 1700 Fribourg, Switzerland; 6 Escola Superior de Saúde da Cruz Vermelha Portuguesa—Lisboa (ESSCVP-Lisboa), 1300-125 Lisboa, Portugal; 7 Laboratory of Molecular Genetics, Instituto de Tecnologia Química e Biológica António Xavier (ITQB), Universidade Nova de Lisboa (UNL), 2780-157 Oeiras, Portugal

**Keywords:** ESBL, carbapenemases, *Escherichia coli*, *Klebsiella pneumoniae*, bivalves, Portugal

## Abstract

Bivalves are filter-feeding organisms and biomarkers of bacterial pollution. Our study aimed to analyze the occurrence and characteristics of extended-spectrum β-lactamase (ESBL)- and carbapenemase-producing *Escherichia coli* among bivalves. A total of 522 bivalve samples were collected along Portuguese shellfish production areas. Homogenized samples were screened for *E. coli* contamination on corresponding selective plates, allowing for concomitant growth of *Klebsiella pneumoniae*. *E. coli* growth was observed in 39% of the samples. Subsequent selective screening identified nine samples (4.4%) contaminated with ESBL producers, corresponding to *E. coli* (*n* = 7) and *K. pneumoniae* (*n* = 2), while a single carbapenemase-producing *K. pneumoniae* (0.5%) was identified. ESBLs were all CTX-M-types commonly identified in human isolates, i.e., CTX-M-32 (*n* = 4), CTX-M-15 (*n* = 4), and CTX-M-14 (*n* = 1). The carbapenemase producer harbored the *bla*_GES-5_ gene located on a ColE plasmid. Clonality was evaluated by multilocus sequence typing, identifying *E. coli* backgrounds as ST10, ST23, ST540, ST617, ST746, SLV206, and SLV2325, commonly identified among environmental and human strains. The *K. pneumoniae* isolates belonged to ST834, ST15, and DLV644. The occurrence of ESBL- and carbapenemase-producing Enterobacteriaceae in bivalves reveals how the marine environment constitutes a reservoir of critical bacterial pathogens, thus potentially representing a risk to human health.

## 1. Introduction

The emergence of extended-spectrum β-lactamase (ESBL)- and carbapenemase-producing Enterobacterales, and their subsequent spread, remains a major global threat. The transmission of such multidrug-resistant bacteria may occur not only from human to human, but also from animal sources to humans via the food chain [1]. In fact, high rates of Enterobacterales producing ESBLs or carbapenemases are seen frequently in food-producing animals, namely in pigs, cattle, and poultry [2]. Although studies reporting ESBL- and carbapenemase-producing Enterobacterales in seafood products are scarce, more frequent surveillance studies have recently been initiated [3,4,5,6,7,8,9,10,11].

Marine bivalves represent a significant proportion of the world’s seafood production sector with a global volume exceeding 15 million tons per year [12]. Due to its privileged geographical position, Portugal combines several characteristics favorable to the exploitation of bivalves: (1) 1793 km of coast under the influence of different currents; (2) a Mediterranean climate in the south and a temperate climate in the north; (3) average water temperatures ranging between 13 and 18 °C; and (4) the existence of several estuaries and lagoons along the coast [13]. The exploitation of bivalves arises from the north to the south of Portugal, with a significant impact on the national economy. 

Bivalve species can be distributed in four major groups: clams, oysters, mussels, and cockles. These organisms feed by filtering phytoplankton and organic matter, a process that allows for the accumulation of various contaminants in these species, including bacteria of both aquatic and anthropogenic origin [14]. Bivalves are therefore good indicators of bacterial, as well as chemical, contamination in a given marine environment. In addition, since bivalves are often eaten raw or lightly cooked, they might constitute a risk for human health.

Bivalve production areas in Europe are assigned to different classes (A, B, or C) according to the content of fecal contamination. Class A areas meet the health standards, whereas class B and C areas require treatment to reduce microbiological contamination before marketing [15]. In Portugal, compliance in bivalve production areas is monitored weekly by the Instituto Português do Mar e Atmosfera (IPMA) and includes the screening for *Escherichia coli* contamination in bivalves collected in all production areas.

According to the European Centre for Disease Prevention and Control data of 2020, multidrug resistance among Enterobacterales remains a major problem in Portuguese hospitals [16]: (a) the mean proportion of ESBL-producing *E. coli* isolates causing human invasive infections was estimated at 14.4%; (b) the prevalence of ESBL producers in invasive *Klebsiella pneumoniae* was estimated at 47.6%; and (c) there has been a notorious, increasing trend in carbapenem resistance among *K. pneumoniae*, reaching 11.6% in 2020. Given this context, it is essential to better understand the sources and modes of dissemination of multidrug-resistant bacteria, especially those involved in human infections, using a global One Health approach, as recommended by the World Health Organization [17]. Following this approach, we have performed surveillance studies on antimicrobial resistance among multidrug-resistant Enterobacterales in different settings, i.e., among hospitalized patients and individuals at the time of hospital admission [18,19], among healthy community individuals (students) [20], among pigs [21], and among wild birds [22]. A single study conducted in 2019 evaluated the bacterial contamination in bivalves collected in Portuguese shellfish farms but did not report any ESBL producers among *Enterobacteriaceae* [23]. Therefore, the aim of the present study was to analyze the occurrence and characteristics of ESBL- and carbapenemase-producing *E. coli* and *K. pneumoniae* among bivalve samples collected in production areas from Portuguese estuaries and coastal waters, using the same prospective methodology as in our previous epidemiological studies.

## 2. Materials and Methods

### 2.1. Bivalve Samples

**Figure 1:**
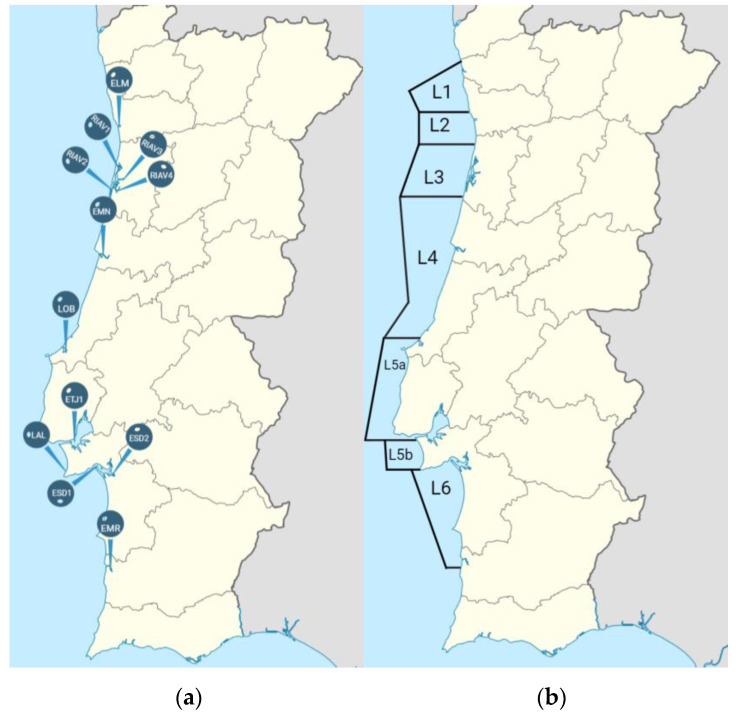
Localization of the 19 shellfish production areas. (**a**) Estuaries, (**b**) Coastal waters. ELM—Estuário do Lima; RIAV1—Ria de Aveiro, Triângulo das Correntes-Moacha; RIAV2—Ria de Aveiro, Canal de Mira; RIAV3—Ria de Aveiro, Canal Principal-Espinheiro; RIAV4—Ria de Aveiro, Canal de Ílhavo; EMN—Estuário do Mondego; LOB—Lagoa de Óbidos; ETJ1—Estuário do Tejo—Jusante Ponte Vasco da Gama; LAL—Lagoa de Albufeira; ESD1—Estuário do Sado, Esteiro da Marateca; ESD2—Estuário do Sado, Canal de Alcácer; EMR—Estuário do Rio Mira; L1—Litoral de Viana; L2—Litoral de Matosinhos; L3—Litoral de Aveiro; L4—Litoral Figueira da Foz—Nazaré; L5a—Litoral Peniche—Cabo Raso; L5b—Litoral Cabo Raso—Cabo Espichel; L6—Litoral Setúbal—Sines.

As part of the national monitoring program for shellfish production area management, 300 batch samples of bivalve mollusks were collected between December 2021 and September 2022 by IPMA from 19 different shellfish production areas along the Portuguese coast (12 estuaries and 7 coastal waters)—Figure 1 and Table 1. A total of 522 bivalve samples were collected.

Each batch sample comprised 10–20 individual bivalve specimens. Approximately 25 g of muscle and intervalvular liquid per bivalve sample were extracted into a sterile container using a scalpel, diluted in maximum recovery diluent (Oxoid, Heysham, Ireland), and homogenized for 60 s using a Stomacher 400 (Seward Laboratory System, London, UK). Homogenized samples were subsequently screened for *E. coli* contamination by plating onto Tryptone Bile X-glucuronide (TBX) selective plates (Oxoid), allowing for concomitant growth of *K. pneumoniae*. A total of 206 TBX plates showing presumptive *E. coli* growth were provided by IPMA for this study.

### 2.2. Bacterial Isolates

The bacterial growth of each TBX plate was resuspended in 3 mL of tryptic soy broth (TSB) (Becton, Dickinson & Co., Franklin Lakes, NJ, USA) for enrichment and incubated at 37 °C overnight. Afterwards, a volume of 25 μL of each culture was inoculated into two selective media: (i) CHROMagar ESBL (Frilabo, Maia, Portugal) for ESBL producers, and (ii) ChromID Carba Smart selective medium (bioMérieux, La Balme-les-Grottes, France) for carbapenem-resistant isolates. For quality control, the selective agar plates were also inoculated with the following control strains: ESBL-positive *K. pneumoniae* ATCC 700603, carbapenemase-positive *K. pneumoniae* ATCC BAA-1705, and ESBL- and carbapenemase-negative *E. coli* ATCC 25922.

The isolates selected for the different media were identified at the species level using the API20E system (bioMérieux).

### 2.3. Antimicrobial Susceptibility Testing

Antimicrobial susceptibility testing was performed on all isolates recovered from the two selective media using the disc diffusion method on Mueller–Hinton (MH) agar plates (Neogen, Lansing, Michigan) for ticarcillin (75 μg), amoxicillin/clavulanic acid (20–10 μg), cefotaxime (30 μg), ceftazidime (10 μg), temocillin (30 μg), cefoxitin (30 μg), ertapenem (10 μg), imipenem (10 μg), meropenem (10 μg), ceftazidime/avibactam (10–4 μg), aztreonam (30 μg), ciprofloxacin (5 μg), trimethoprim-sulfamethoxazole (SXT) (1.25–23.75 μg), tetracycline (30 μg), amikacin (30 μg), gentamicin (15 μg), and tobramycin (10 μg) (Bio-Rad Laboratories, Algés, Portugal), following EUCAST recommendations and breakpoint tables. Susceptibility to fosfomycin was evaluated by the disk diffusion method (50 μg) on MH agar plates supplemented with 25 μg/mL glucose-6-phosphate, according to EUCAST guidelines [24]. Strain *E. coli* ATCC 25922 was used for quality control. Multidrug resistance was defined as acquired non-susceptibility to at least one agent in three or more antimicrobial categories [25].

### 2.4. Molecular Analysis

Identification of ESBL and carbapenemase genes was performed by PCR as previously reported [26,27]. All positive amplicons were sent out for sequencing (Eurofins Genomics, Ebersberg, Germany).

The clonal relationship of the ESBL- and carbapenemase-producing isolates was evaluated by multilocus sequence typing (MLST) [28], and sequence types (STs) were assigned using the MLST databases for *E. coli* (http://enterobase.warwick.ac.uk/species/ecoli/allele_st_search; accessed on 6 December 2022) and *K. pneumoniae* (https://bigsdb.pasteur.fr/klebsiella/; accessed on 6 December 2022).

### 2.5. Conjugation Experiments and Plasmid Analysis

Mating-out assays were performed using the azide-resistant *E. coli* J53 as the recipient. *E. coli* J53 and the *bla*_GES-5_-positive donor were separately inoculated into 5 mL of tryptic soy broth (TSB (Frilabo)) and incubated at 37 °C for 5 h. The samples were subsequently mixed at a ratio of 1:4 (200 µL donor:800 µL recipient), centrifuged for 1 min at 3500 rpm, and 800 µL of the supernatant were discarded. The pellet was resuspended in the remaining 200 µL. This volume was deposited onto 22 µm filters onto a tryptic soy agar (TSA (Frilabo)) plate and incubated for 3 h. After 3 h, the filter was resuspended in 5 mL of 0.9% NaCl, and 100 µL of the mixture were plated onto TSA agar plates supplemented with cefoxitin (50 μg/mL) and azide (100 μg/mL). Susceptibility testing was performed for the *E. coli* transconjugants, and positivity for *bla*_GES-5_ was assessed by PCR.

The plasmid harboring the GES-5 carbapenemase-encoding gene was classified according to its incompatibility group using the PCR-based replicon typing (PBRT) method performed on DNA recovered from *E. coli* transconjugants, as described previously [29].

### 2.6. Statistical Analysis

Statistical analysis was performed using GNU R v 4.0.3 under RStudio 2022.07.1 + 554. Fisher’s independence test was used to identify variables associated with the *E. coli* contamination and carriage of ESBL and carbapenemase producers. All *p*-values below 0.05 were considered statistically significant.

## 3. Results

### 3.1. Population Description

Out of the 522 bivalve samples collected during a nine-month period, 206 (39%) grew *E. coli* on the TBX selective plates. Those 206 bivalve samples were recovered from the 19 production areas included in the study (Table 1). Of note, *E. coli* contamination rates were over 40% in 11 of the 19 production areas, all but one (L5b) being located in estuaries. In contrast, the production areas with lower contamination rates (<15%) were all situated in coastal waters (*p* < 0.001). However, the two coastal production areas closest to the Lisbon metropolitan area (L5a/b and L6) showed a significant amount (30–42%) of *E. coli* contamination.

*E. coli* isolates were detected in 18 (out of the 19) bivalve species (Table 2). Four species showed higher rates (>50%) of contamination: *Venus casina* (1/1; 100%), *Scrobicularia plana* (10/15; 67%), *Dosinia exoleta* (3/5; 60%), and *Solen marginatus* (29/57; 51%). Only the species *Scrobicularia plana* showed a statistically significant result (*p* = 0.03).

### 3.2. Carriage of Multidrug-Resistant Enterobacterales

Out of the 206 samples recovered on the TBX plates, nine enterobacterial isolates grew on the CHROMagar ESBL agar, and one isolate grew on the ChromID Carba Smart selective medium.

The antimicrobial susceptibility testing of the nine former isolates showed non-susceptibility to ticarcillin, cefotaxime and temocillin (100%), ceftazidime and aztreonam (*n* = 8; 89%), tetracycline (*n* = 6; 66%), amoxicillin/clavulanic acid (*n* = 4; 44%), ciprofloxacin, SXT and tobramycin (*n* = 3; 33%), gentamicin (*n* = 2; 22%), and amikacin (*n* = 1; 11%) (Table 3). All isolates remained susceptible to cefoxitin, ertapenem, imipenem, meropenem, and fosfomycin. The single isolate growing on the selective medium for carbapenem resistance showed non-susceptibility to amoxicillin/clavulanic acid, ceftazidime, temocillin, cefoxitin, ertapenem, imipenem, meropenem, and tobramycin. None of the 10 isolates were resistant to the newly developed ceftazidime/avibactam combination. Half of the isolates presented resistance to at least two antibiotic classes other than β-lactams and were considered multidrug-resistant.

Nine bivalve samples out of the 206 that grew on the TBX plates carried an ESBL producer (9/206; 4.4%), and one was contaminated by a carbapenemase producer (1/206; 0.5%)—Table 1. The 10 isolates belonged to two species: *E. coli* (*n* = 7; 70%) and *K. pneumoniae* (*n* = 3; 30%). All ESBLs were from the CTX-M-type, with CTX-M-32 and CTX-M-15 being the most frequently found (*n* = 4; 40% each), followed by CTX-M-14 (*n* = 1). The single carbapenemase producer (*K. pneumoniae*) harbored *bla*_GES-5_.

The ESBL-producing isolates were recovered from six production areas (Table 1), out of which two showed higher frequencies of ESBL producers (*p* < 0.01 in both cases): ELM (22%) and ESD2 (17%)—Figure 1. The single carbapenemase-producing isolate was found in EMN. All ESBL- and carbapenemase-producing isolates were harvested from estuaries, although this result was not statistically significant (*p* = 0.16).

The nine ESBL-producing Enterobacterales were isolated from five different bivalve species (Table 2): *Venerupis corrugate* (*n* = 2), *Magallana gigas* (*n* = 2), *Magallana angulata* (*n* = 2), *Scrobicularia plana* (*n* = 1), and *Solen marginatus* (*n* = 2), whereas the carbapenemase-producing isolate was recovered from *Cerastoderma edule*. Four (25%) ESBL-producing isolates were recovered from oysters (*Magallana gigas* and *Magallana angulata*), although this result was not statistically significant (*p* = 0.057).

The MLST analysis showed high heterogeneity among the seven ESBL-producing *E. coli* isolates that were distributed into seven different clones; although two isolates, belonging to ST617 (CTX-M-32) and ST10 (CTX-M-15), were classified into the same clonal complex (CC), CC10—Table 3. The two ESBL-producing (CTX-M-15) *K. pneumoniae* isolates belonged to ST834 and ST15, while the GES-5 carbapenemase producer was classified as a double-locus variant (DLV) of ST644. The mating-out assay followed by PBRT revealed that the *bla*_GES-5_ gene was located on a ColE plasmid.

## 4. Discussion

Here we present a comprehensive study on the occurrence and characteristics of ESBL- and carbapenemase-producing isolates among bivalve samples, during a nine-month period, from different shellfish production areas in Portuguese estuaries and coastal waters. Our study revealed that a high proportion (39%) of bivalves were contaminated with *E. coli*, and among these, 4% were contaminated with ESBL producers. In addition, one sample was contaminated with a carbapenemase-producing *K. pneumoniae*. Of note, all ESBL- and carbapenemase-producing isolates were harvested from estuaries, where effluent flow is less affected by tides and there is lower salinity compared to coastal waters.

The rates of ESBL-producing isolates in the present study were slightly higher than those reported among bivalves in other countries: (i) a study on antibiotic resistance of *E. coli* from marine bivalves collected in Norway identified ESBL-encoding genes in 1% of isolates (2/199) [5], and (ii) 1.6% of clams bought from retail markets in Tunisia were contaminated with ESBL-producing Enterobacterales [9]. In contrast, the proportion was much lower than the 15% (21/141) reported among *E. coli* isolates recovered from clams in Italy [6] and India, where 53% of fresh shellfish from retail markets exhibited ESBL-producing *E. coli* isolates [11].

All ESBLs found in the present study belonged exclusively to the CTX-M family, i.e., CTX-M-32, CTX-M-15, and CTX-M-14. These enzymes are commonly identified in human isolates worldwide, including Portugal [19,20]. Accordingly, we may speculate that the ESBL producers identified among bivalves might correspond to strains colonizing the human gut.

One out of the three *K. pneumoniae* identified in the present study displayed a carbapenemase gene, which is usually much less frequent among bivalve samples than are ESBLs. Sporadic carbapenem-producing Enterobacterial isolates were previously reported: In Germany, one VIM-1-producing *E. coli* was isolated from clams collected in Italy [10]. In Tunisia, two *K. pneumoniae* isolates (*bla*_NDM-1_ and *bla*_OXA-48_) were isolated in clams, and a KPC-3-producing *E. coli* was recovered in mussels purchased in retail markets [8,9]. In Myanmar, two NDM-1 producers (one *E. coli* and one *K. pneumoniae*) were isolated from a clam and a prawn, respectively [30], and in Canada, three and two clam samples imported from Vietnam contained *E. cloacae* harboring *bla*_IMI-1_ and *bla*_NDM-1_, respectively [31]. We identified in the present study the first enterobacterial isolate producing a carbapenemase of GES-type (GES-5) recovered from bivalve samples. This carbapenemase has been previously detected in Portugal among humans [18], gulls [22], the aquatic environment [32], and now bivalves.

The seven ESBL-producing *E. coli* strains belonged to distinct genetic backgrounds (seven STs), which suggests multiple sources of *E. coli* contamination in the harvesting production areas. Most of these STs have been previously found among humans and/or animals and/or in the environment, namely in Portugal. In fact, ST10 was the predominant clone among *E. coli* recovered from healthy students in Lisbon [20], was frequently isolated in Portuguese hospitals [33], and was found among healthy and sick cats in the country [34]. Moreover, ST10 was the predominant lineage among *E. coli* present in irrigation water and vegetables from 16 household farms in Portugal [35]. Concerning bivalves, one *E. coli* ST10 producing VIM-1 was isolated from a clam harvested in the Mediterranean Sea (Italy) [10]. ST617, which also belongs to CC10, has been formerly reported in Portugal in a CTX-M-55 *E. coli* strain colonizing a healthy dog [36] and from two CTX-M-15 *E. coli* strains isolated from the effluent from a wastewater treatment plant [37]. In addition, ST617 has been previously described in a CTX-M-14 *E. coli* isolate from clams sold in Tunisian markets [9].

In the present study, one *E. coli* isolate belonged to a single-locus variant (SLV) of ST206. This ST has been reported only once in Portugal, corresponding to a single CTX-M-15 isolate recently recovered from a pigeon fecal sample in Lisbon [38]. ST206 is mainly associated with animals, being the main clone among chickens in Nigeria [39] and frequently found among food-chain animals in China [40,41]. Concerning *E. coli* ST23, it has also been previously reported in Portugal among hospital outpatients [33]. This ST was frequently found among human isolates, namely causing urinary tract infections (UTIs) in nursing homes in the Netherlands [42] and also causing mastitis in cattle in France [43].

One ESBL-producing *K. pneumoniae* strain found in the present study belonged to ST15. ST15 was the main (32%) clonal type among 509 *K. pneumoniae* isolates collected nationwide in Portugal from 16 hospitals and environmental settings between 1980 and 2019 [44]. ST15 includes clinical, animal, and environment isolates, being an international lineage with a wide geographic distribution [45]. Overall, the clinically relevant bacteria identified in our study seem to qualitatively mirror the human panorama, not only in terms of β-lactamases circulating in this specific environment, but also in terms of clonal backgrounds. These results suggest a very likely human fecal contamination, possibly through sewage effluents that are discharged in estuaries where bivalve production areas are located.

In conclusion, this study constitutes the first report of enterobacterial ESBL and carbapenemase producers among bivalves in Portuguese coastal waters. Although a relatively low occurrence of ESBL and carbapenemase producers was detected, our study demonstrates that bivalves collected for human consumption may act as a potential reservoir of multidrug-resistant bacterial pathogens that eventually may be transmitted through the food chain. Therefore, in the future, the compliance of bivalve production areas routinely monitored by IPMA should also include screening for ESBL- and carbapenemase-producing Enterobacterales contamination in bivalves collected in the different production areas.

## Figures and Tables

**Table 1:** ESBL and carbapenemase producers by bivalve production area.

Shellfish Production Area		No. of Screenings	Samples Recovered	Samples with *E. coli* Contamination ^1^	ESBL Producers ^2^	Carbapenemase Producers ^2^
Estuaries (*n* = 12)	ELM	12	22	9 (41%)	2 (22%)	-
	RIAV1	29	51	23 (45%)	1 (4%)	-
	RIAV2	23	35	15 (43%)	-	-
	RIAV3	20	33	13 (39%)	1 (8%)	-
	RIAV4	18	32	14 (44%)	-	-
	EMN	18	38	20 (53%)	1 (5%)	1 (5%)
	LOB	10	58	25 (43%)	-	-
	ETJ1	19	28	14 (50%)	-	-
	LAL	11	19	7 (37%)	-	-
	ESD1	25	27	12 (44%)	1 (8%)	-
	ESD2	17	36	18 (50%)	3 (17%)	-
	EMR	8	16	4 (25%)	-	-
Coastal waters (*n* = 7)	L1	14	14	1 (7%)	-	-
	L2	12	16	1 (6%)	-	-
	L3	11	11	1 (9%)	-	-
	L4	7	7	1 (14%)	-	-
	L5a	10	10	3 (30%)	-	-
	L5b	18	24	10 (42%)	-	-
	L6	18	45	15 (33%)	-	-
Total	19	300	522	206 (39%)	9 (4.4%)	1 (0.5%)

^1^ The percentage is relative to the number of samples recovered. ^2^ The percentage is relative to the number of samples with *E. coli* contamination. ELM—Estuário do Lima; RIAV1—Ria de Aveiro, Triângulo das Correntes-Moacha; RIAV2—Ria de Aveiro, Canal de Mira; RIAV3—Ria de Aveiro, Canal Principal-Espinheiro; RIAV4—Ria de Aveiro, Canal de Ílhavo; EMN—Estuário do Mondego; LOB—Lagoa de Óbidos; ETJ1—Estuário do Tejo—Jusante Ponte Vasco da Gama; LAL—Lagoa de Albufeira; ESD1—Estuário do Sado, Esteiro da Marateca; ESD2—Estuário do Sado, Canal de Alcácer; EMR—Estuário do Rio Mira; L1—Litoral de Viana; L2—Litoral de Matosinhos; L3—Litoral de Aveiro; L4—Litoral Figueira da Foz—Nazaré; L5a—Litoral Peniche—Cabo Raso; L5b—Litoral Cabo Raso—Cabo Espichel; L6—Litoral Setúbal—Sines.

**Table 2:** ESBL and carbapenemase producers by bivalve species.

Bivalve Species		Samples Recovered ^1^	Samples with *E. coli* Contamination ^2^	ESBL Producers ^3^	Carbapenemase Producers ^3^
Clam	*Venerupis corrugata*	43 (8%)	21 (49%)	2 (10%)	-
Clam	*Ruditapes philippinarum*	38 (7%)	17 (45%)	-	-
Clam	*Ruditapes decussatus*	33 (6%)	14 (42%)	-	-
Clam	*Spisula solida*	31 (6%)	3 (10%)	-	-
Clam	*Scrobicularia plana*	15 (3%)	10 (67%)	1 (10%)	-
Clam	*Donax trunculus*	11 (2%)	4 (36%)		-
Clam	*Venus verrucosa*	9 (2%)	2 (22%)		-
Clam	*Dosinia exoleta*	5 (1%)	3 (60%)	-	-
Clam	*Callista chione*	4 (1%)	-	-	-
Clam	*Venus casina*	1 (<1%)	1 (100%)		-
Razor clam	*Solen marginatus*	57 (11%)	29 (51%)	2 (7%)	-
Razor clam	*Ensis siliqua*	16 (3%)	7 (44%)	-	-
Oyster	*Magallana gigas*	52 (10%)	24 (46%)	2 (8%)	-
Oyster	*Magallana angulata*	28 (5%)	11 (39%)	2 (18%)	-
Oyster	*Ostrea edulis*	9 (2%)	2 (22%)	-	-
Cockle	*Cerastoderma edule*	82 (16%)	33 (40%)	-	1 (3%)
Cockle	*Glycymeris glycymeris*	14 (3%)	1 (7%)	-	-
Cockle	*Laevicardium crassum*	3 (1%)	1 (33%)	-	-
Mussel	*Mytilus galloprovincialis*	71 (14%)	23 (32%)	-	-
Total	19	522	206 (39%)	9 (4%)	1 (0.5%)

^1^ The percentage is relative to the total number of samples recovered. ^2^ The percentage is relative to the number of samples of the bivalve species recovered. ^3^ The percentage is relative to the number of samples with *E. coli* contamination.

**Table 3:** Characteristics of the ESBL- and carbapenemase-producing isolates.

8	Isolate	ST	CC	ESBL/ Carbapenemase	TIC	AMC	CTX	CZD	TEM	FOX	CZA	ETP	IPM	MEM	ATM	CIP	SXT	TET	AKN	GMI	TMN	FOS
*E. coli*	R6129	23	23	CTX-M-15	R	R	R	R	I	S	S	S	S	S	I	S	S	R	S	S	S	S
*E. coli*	R6130	746	ND	CTX-M-32	R	S	R	R	I	S	S	S	S	S	R	S	S	R	S	S	S	S
*E. coli*	R6131	SLV206	206	CTX-M-14	R	S	I	S	I	S	S	S	S	S	S	S	S	S	S	S	S	S
*E. coli*	R6132	SLV2325	ND	CTX-M-32	R	S	R	R	I	S	S	S	S	S	R	S	S	S	S	S	S	S
*E. coli*	R6133	617	10	CTX-M-32	R	S	R	R	I	S	S	S	S	S	I	R	R	R	S	S	S	S
*E. coli*	R6134	10	10	CTX-M-15	R	S	R	I	I	S	S	S	S	S	R	I	R	R	S	S	S	S
*E. coli*	R6136	540	ND	CTX-M-32	R	R	R	R	R	S	S	S	S	S	R	S	S	R	S	R	R	S
*K. pneumoniae*	R6128	834	ND	CTX-M-15	R	R	R	R	I	S	S	S	S	S	R	S	S	R	R	R	R	S
*K. pneumoniae*	R6135	15	ND	CTX-M-15	R	R	R	R	I	S	S	S	S	S	R	R	R	S	S	S	R	S
*K. pneumoniae*	R6137	DLV644	ND	GES-5	R	R	S	R	I	R	S	R	I	I	S	S	S	S	S	S	S	S

ST—Sequence type determined by multilocus sequence typing. SLV—Single-locus variant. DLV—Double-locus variant. CC—Clonal complex. ND—Not determined. TIC—Ticarcillin; AMC—Amoxicillin/clavulanic acid; CTX—Cefotaxime; CZD—Ceftazidime; TEM—Temocillin; FOX—Cefoxitin; CZA—Ceftazidime/avibactam; ETP—Ertapenem; IMP—Imipenem; MEM—Meropenem; ATM—Aztreonam; CIP—Ciprofloxacin; SXT—Trimethoprim-sulfamethoxazole; TET—Tetracycline; AKN—Amikacin; GMI—Gentamicin; TMN—Tobramycin.

## Data Availability

The data presented in this study are all available in the main text.
